# Identifying and characterizing lincRNA genomic clusters reveals its cooperative functions in human cancer

**DOI:** 10.1186/s12967-021-03179-5

**Published:** 2021-12-14

**Authors:** Hanxiao Zhou, Yue Gao, Xin Li, Shipeng Shang, Peng Wang, Hui Zhi, Shuang Guo, Dailin Sun, Hongjia Liu, Xia Li, Yunpeng Zhang, Shangwei Ning

**Affiliations:** College of Bioinformatics Science and Technology, Harbin Medical University, Harbin, 150081 Heilongjiang China

**Keywords:** LincRNA, Chromosome physical location, Clustering, Coordinated regulation, Pan cancer, Clinical prognosis

## Abstract

**Background:**

Emerging evidence has revealed that some long intergenic non-coding RNAs (lincRNAs) are likely to form clusters on the same chromosome, and lincRNA genomic clusters might play critical roles in the pathophysiological mechanism. However, the comprehensive investigation of lincRNA clustering is rarely studied, particularly the characterization of their functional significance across different cancer types.

**Methods:**

In this study, we firstly constructed a computational method basing a sliding window approach for systematically identifying lincRNA genomic clusters. We then dissected these lincRNA genomic clusters to identify common characteristics in cooperative expression, conservation among divergent species, targeted miRNAs, and CNV frequency. Next, we performed comprehensive analyses in differentially-expressed patterns and overall survival outcomes for patients from The Cancer Genome Atlas (TCGA) and The Genotype-Tissue Expression (GTEx) across multiple cancer types. Finally, we explored the underlying mechanisms of lincRNA genomic clusters by functional enrichment analysis, pathway analysis, and drug-target interaction.

**Results:**

We identified lincRNA genomic clusters according to the algorithm. Clustering lincRNAs tended to be co-expressed, highly conserved, targeted by more miRNAs, and with similar deletion and duplication frequency, suggesting that lincRNA genomic clusters may exert their effects by acting in combination. We further systematically explored conserved and cancer-specific lincRNA genomic clusters, indicating they were involved in some important mechanisms of disease occurrence through diverse approaches. Furthermore, lincRNA genomic clusters can serve as biomarkers with potential clinical significance and involve in specific pathological processes in the development of cancer. Moreover, a lincRNA genomic cluster named Cluster127 in *DLK1-DIO3* imprinted locus was discovered, which contained MEG3, MEG8, MEG9, MIR381HG, LINC02285, AL132709.5, and AL132709.1. Further analysis indicated that Cluster127 may have the potential for predicting prognosis in cancer and could play their roles by participating in the regulation of PI3K-AKT signaling pathway.

**Conclusions:**

Clarification of the lincRNA genomic clusters specific roles in human cancers could be beneficial for understanding the molecular pathogenesis of different cancer types.

**Supplementary Information:**

The online version contains supplementary material available at 10.1186/s12967-021-03179-5.

## Introduction

Long intergenic non-coding RNAs (lincRNAs) are defined as RNA transcripts longer than 200 nucleotides that do not overlap with the exons of any protein-coding gene [1]. Acting mechanisms of lincRNAs are complex such as chromatin remodeling, co-transcriptional regulation, scaffolding of nuclear or cytoplasmic complexes, and interacting with other cellular factors [1]. Dysregulation of lincRNAs involves in human cancers [2]. It has been found that difference in lincRNA profiling between normal and cancer cells is strongly associated with cancer progression [3]. Recent studies have revealed lincRNA can be potential therapeutic targets or prognostic biomarkers for various types of cancer [4]. Several molecular mechanisms of lincRNAs have been described relatively clearly. For example, loss of HOTAIR can inhibit cancer invasiveness [5]. MALAT1 plays an important role in cancer and metastasis by regulation of gene expression or alternative splicing [6]. However, little is known about the regulatory mechanisms of most lincRNAs especially their interaction in normal physiology and tumor development.

Recent studies revealed that lincRNAs may act as modular scaffolds for protein–chromatin interactions [7]. In this way, lincRNAs contain discrete domains that interact with specific protein complexes [8], bringing proteins together in a functional complex [9]. Moreover, a novel RNA chromosome conformation capture (R3C) strategy demonstrated lincRNA can regulate genes in cancer [10, 11], suggesting lincRNAs may regulate biological processes synergistically [12]. For example, HOTTIP and HOXA13 facilitated tumorigenesis and metastasis by acting as a synergistic role [13, 14]. In addition, a few studies have suggested a substantial fraction of lincRNA can influence the expression of neighboring genes, both lncRNAs and mRNAs [15]. Moreover, a cluster of lncRNAs in neighboring genomic locations may co-mediate pathophysiological mechanisms [16]. For instance, a recent study identified a series of lincRNAs called Eleanors (ESR1 locus enhancing and activating noncoding RNAs) that activates the ESR1 locus in breast cancers [17]. Wang et al. noticed that a series of clusters localized close together involved in proper growth, development, and visual activities [16]. However, the varied roles of lincRNA genomic clusters in human pan-cancer have not been globally studied in a systematic manner.

A gene cluster consists of closely spaced genes on a chromosome and is frequently co-expressed to accomplish a function or a group of related functions [14, 18, 19]. Aberrant DNA methylation of HOXA gene cluster is closely linked to the loss of expression in breast cancer [20]. In recent years, several types of small non-coding RNA (ncRNA) clusters have been studied in more detail. Several studies have suggested a snoRNA cluster containing SNORD115 (H/MBII-52) and SNORD116 (H/MBII-85) at the imprinted Prader-Willi locus, the cluster played key roles in the etiology of the Prader-Willi Syndrome (PWS) [21]. In addition, miRNA clusters can be biomarkers for diagnosis, prognosis, and targeted therapy [22]. Clustered miRNAs tend to be evolutionarily conserved and co-expressed to coordinately regulate functionally related genes [23]. For instance, the miR-17-92 miRNA cluster drives metabolic reprogramming downstream of Myc to regulate tumor metabolism [24]. Several computational methods for miRNA cluster identification recently have been developed. Altuvia et al. identified miRNA clusters using a computation algorithm at different maximum inter-miRNA distances (MIDs) and utilized the FP-growth algorithm to efficiently discover the conserved co-occurrence of miRNA clusters [25]. However, computational methods to identify the clusters of lincRNAs have not seen much success.

Here, we constructed a computational algorithm basing a sliding window approach to identify lincRNA genomic cluster on a chromosome. Common characteristics were observed within lincRNA genomic clusters identified by our study. LincRNAs in clusters were found to have functional properties as evidenced by expression level, evolutionary conservation, density of miRNA target sites, and similar CNV frequency. Then, we performed a systematic pan-cancer analysis of 14 cancer types to identify the “common” clusters and “unique” clusters. Moreover, we identified some lincRNA genomic clusters and these core clusters could distinguish between two groups of patients with different clinical outcomes among different cancer types. Functional analysis revealed that a set of clusters are participating in the cancer-related functions. In conclusion, the present study highlighted the effect of lincRNA genomic clusters in human cancers, which revealed their possibility as novel biomarkers and treatment targets in cancer.

## Materials and methods

### Human lincRNA location data and gene annotation data

The intergenic long non-coding RNA (lincRNA) location data and protein-coding genes annotation data were downloaded from Ensembl Genome Browser database (http://www.ensemblgenomes.org, Ensembl v92, GRCh38.p12), including gene stable ID, Chromosome/scaffold name, Gene start (bp), Gene end (bp), Strand, and Gene name.

### Sources and scope of cancer and normal data

We obtained lincRNA expression data on 20 cancer types from TANRIC (https://ibl.mdanderson.org/tanric/_design/basic/index.html) [26]. Protein coding gene expression data and clinical data on these 20 cancers were acquired from the TCGA database using the UCSC Xena browser (http://xena.ucsc.edu/). To compare tumor samples to healthy tissues, GTEx [27] samples were downloaded from the UCSC Xena browser. The number of normal and cancer samples from TCGA and GTEx is shown in Additional file 2: Table S1.

### miRNA-lincRNA interaction data

To ensure high quality of miRNA-lincRNA interaction, we obtained high-throughput experimentally verified miRNA-lincRNA interaction data by collecting lincRNAs from four databases (10,385 miRNA-lincRNA interaction from LncBase v.2 [28], 11,921 miRNA-lincRNA interaction from RAID v2 [29], 1140 miRNA-lincRNA interaction from miRTarBase [30], 121 miRNA-lincRNA interaction from TarBase v7.0 [31]). To select bona fide targets, 22,690 miRNA-lincRNA interactions identified using the four databases were integrated into a comprehensive data set, containing 1627 miRNAs.

### Computing the lincRNA genomic cluster

Nearest neighbor distances between consecutive lincRNA genes on each chromosome were computed. In this study, we presented an algorithm for the discovery of lincRNA genomic clusters from the genomic location datasets by a sliding window approach. Those sliding windows should meet the two criteria as follows: (a) the distance between consecutive lincRNAs should be less than the defined distance threshold; (b) the lincRNAs in a cluster should be more than four. We identified a lincRNA genomic cluster using a threshold value to start extending downstream from the first lincRNA on each chromosome. When the adjacent distances between lincRNAs were less than the threshold user-defined, the four adjacent lincRNAs are classified into a candidate cluster and then extent a downstream lincRNA to determine whether the above criteria are met, then continue to repeat extension until it does not meet the criteria; Otherwise, the current extension step will stop and the last lincRNA of the cluster will be defined as the closing cluster position.

To evaluate the statistical significance of the lincRNA clustering for each cluster, we chose a random start for each lincRNA of the cluster on the same chromosome and expanded downward to the same number of lincRNAs as in the real cluster. We defined the distance between the start site of the first lincRNA and the end site of the last lincRNA in a cluster as the cluster distance. Then, we computed the cluster distance of the random lincRNA genomic cluster and checked whether the cluster distance of the random lincRNA genomic cluster is lower than that of the lincRNA genomic cluster. This procedure was repeated 1000 times. The fraction of times for which the random cluster distances were smaller than the real cluster distance provides the statistical significance for the lincRNA clustering. P-value < 0.005 was determined and employed to identify the statistical significance of the lincRNA genomic cluster. We repeated this procedure at different distance thresholds (15 kb, 20 kb, 25 kb, 30 kb, 35 kb, 40 kb, 45 kb, 50 kb, 55 kb, 60 kb, 65 kb, 70 kb, 75 kb, and 80 kb) and compared the clustering of the lincRNA genes.

### Calculating the conservation of lincRNA genomic cluster

To measure the conservation of lincRNAs in clusters of sequences across multiple species, conservation scores were generated for each lincRNA in clusters and out of clusters. We used UCSC phastCons conservation score and phylogenetic P values (phyloP) conservation score which for the human genome (hg38) calculated from multiple alignments with other 99 vertebrate species to get the conservation level of lincRNA. We defined the conservation of lincRNA genomic clusters as the average phastCons and average phyloP conservation scores of lincRNAs in the cluster.

### Co-expression analysis of lincRNA genomic clusters in different cancer types

The expression values of lincRNAs were obtained as described above. Co-expression of lincRNA genomic clusters were identified in different cancer with Pearson's correlation coefficient tests and a hypergeometric distribution-based test. A cluster that two criteria were satisfied is expected to co-expression.

#### Construction of the WGCNA co-expression network

WGCNA [32] was an algorithm used in gene co-expression network identification by high-throughput cancer expression profiles in different cancer types. In our study, the one-step function was used for network construction and detection of consensus modules. Soft-thresholding power and module size cut-off 30 were chosen as the threshold to identify co-expressed gene modules. And the threshold for merging modules was set to 0.25 as default. In an attempt to detect expression properties of lincRNA genomic cluster, a hypergeometric test was employed in our study, which could evaluate the significance of the co-expression of lincRNA in each cluster. P-values were calculated for lincRNA genomic clusters enriched on each co-expression module in different cancer using the hypergeometric distribution. The genome owned K lincRNAs, of which M and N were the number of lincRNAs related to the present co-expression module and cluster, and Y was the shared lincRNA number of co-expression module and cluster. The P-value was calculated using the following equation:

$$\mathrm{P}=1-\sum_{t=0}^{Y}\frac{\left(\genfrac{}{}{0pt}{}{M}{t}\right) \left(\genfrac{}{}{0pt}{}{K-M}{N-t}\right)}{\left(\genfrac{}{}{0pt}{}{K}{N}\right)}$$

To identify cluster-related modules, for each module and each lincRNA genomic cluster, we used a hypergeometric test, and P-value ≤ 0.05 was used as the significance threshold. Then, the cluster-related module should be enriched with at least two lincRNAs of cluster and the P-value of the module should be the lowest. Then, for each lincRNA genomic cluster with a cluster-related module we performed 1000 times’ random sampling the same number of lincRNAs in the cluster and repeated the above process, the procedure set the threshold of permutation test q value was 0.05.

#### Co-expression measure for gene pairs in the lincRNA genomic cluster

For each lincRNA genomic cluster, we computed the correlation coefficient for 20 cancer types:

$${\text{m}}{R}_{c}\left(T\right)=mean\sum _{j=1,k=1}^{L}\left|Cor\left({Exp}_{j },{Exp}_{k}\right)\right|$$

where $Cor\left({Exp}_{j },{Exp}_{k}\right)$ is the Pearson's correlation coefficient between the tumor samples expressions of ${\mathrm{lincRNA}}_{j}$ and ${\mathrm{lincRNA}}_{k}$ in the cluster, these relationships describe the similarity between expression patterns of the lincRNA pair across all the tumor samples. And L is the number of lincRNA in the cluster.

For each lincRNA genomic cluster we performed 1000 times’ random sampling the same number of lincRNAs in cluster and computed the correlation coefficient in 20 cancer samples, procedure set the threshold of permutation test P-value was 0.05.

### Copy number variation (CNV) data and CNV analysis

We obtained the level-3 copy number variation (CNV) data of Affymetrix SNP 6.0 array for 20 cancer types from The Cancer Genome Atlas (TCGA) data portal. Copy number variation region was identified by GISTIC 2.0 [33]. We calculated the CNV frequency in each cancer type with CNV amplification and deletion.

### Differential expression analysis of lincRNA genomic clusters

As a subset of tumor samples did not have any matched adjacent tissue samples, we used those cancers that have adjacent normal tissues in TCGA. Each expression value is converted to the log base 2 of the value. R package limma was used to identify significant differentially expressed lincRNAs in tumor and normal samples of different cancers. Absolute P-value < 0.01 were considered significant. If more than half of lincRNAs in the cluster were differentially expressed across cancers, we defined the lincRNA genomic cluster as co-differentially-expressed. For comparison of GTEx normal tissues and TCGA tumor tissues, differential expression analysis was performed using limma.

### Hierarchical clustering based on single-sample gene set enrichment (ssGSEA)

In order to better detect and characterize the lincRNA genomic clusters shared among tumors, we applied ssGSEA to bulk RNA-Seq data from tumor samples. We assessed ssGSEA score (based on Euclidean distance and KM cluster algorithm) for each cluster among the 20 cancer types using R package GSVA [34]. We calculated average ssGSEA lincRNAs genomic cluster score for each cancer and consensus clustering was performed by the ConsensusClusterPlus R package [35] to identify modules. Next, we applied the lncSEA tool to dissect the function of modules [36].

### Survival analysis

Univariable Cox regression and Kaplan–Meier survival analyses were used to analyze the prognostic significance of the each lincRNAs in clusters. Next, Multivariate Cox regression analyses were used to construct and analyze risk scores based on lincRNAs of each lincRNA genomic cluster. According to the median risk score, patients were classified into low- and high-risk groups. The overall survival (OS) was compared between the groups based on Kaplan–Meier curves. All analyses were performed within the R 2.4.3 framework.

### Correlation analysis between lincRNAs and mRNAs

Pearson's correlation coefficient (PCC) was calculated between expressed values of each lincRNA and mRNA in normal samples and tumor samples. For the mRNAs with absolute value of PCC not less than 0.35 were selected to functional enrichment.

### Functional annotation of the lincRNA genomic cluster

Functional annotation was performed for each lincRNA genomic cluster with the Enrichr tool [37] online web server using default parameters. We obtained enriched Gene Ontology (GO) terms, KEGG pathways, Connectivity Map (CMAP) drugs. To determine potential functions of lincRNAs in clusters, co-expressed mRNAs for the lincRNAs were obtained. Then Gene Ontology (GO) and KEGG enrichment analyses were implemented to identify the affected the pathways involved in and the potential functions of lincRNA co-expressed mRNAs using the ClusterProfiler [38]. P-value < 0.05 was considered to indicate a statistically significant difference.

## Result

### Constructing landscape of lincRNA genomic clusters across tumor types

Gene order is not random in eukaryotic chromosomes, and co-regulated genes tend to be clustered [39]. We proposed a computational algorithm basing a sliding window approach to determine whether lincRNAs are also clustered on the chromosome. Then we demonstrated a pan-cancer analysis workflow to characterize lincRNA genomic clusters (Additional file 1: Fig. S1). Using 1000 random permutations with a P-value of 0.005, we identified significant lincRNA genomic clusters at different distances. The chromosome distribution of lincRNA genomic clusters at different distances is presented in Additional file 1: Fig. S2.

**Fig. 1:**
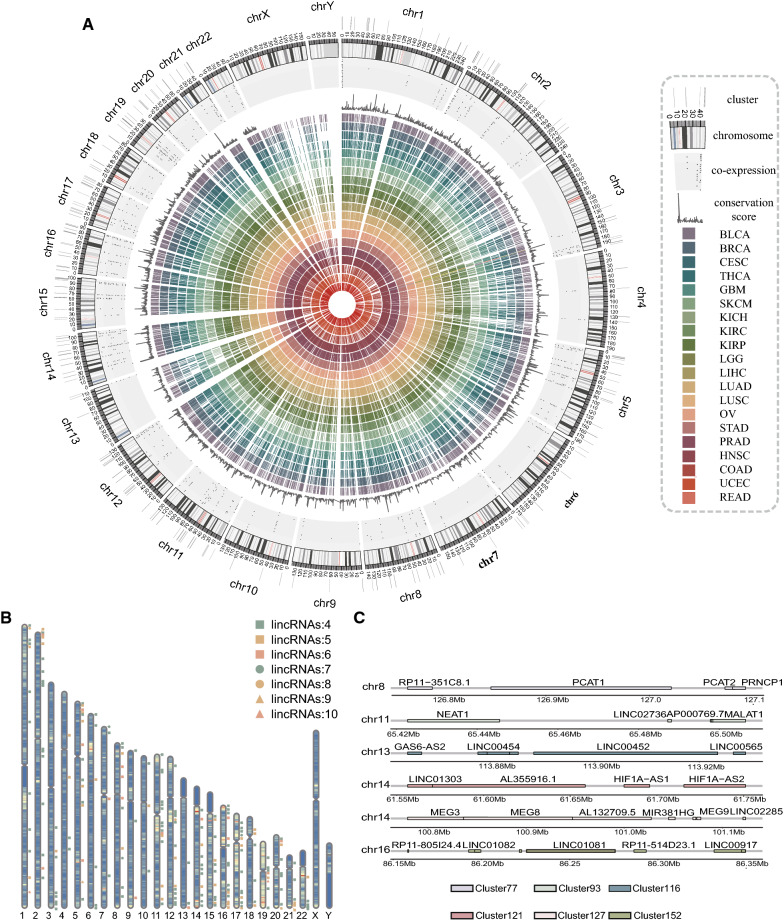
The Global landscape of lincRNA genomic clusters in human cancers. (**A**) Global map of lincRNA expression, co-expression of lincRNA genomic clusters across cancer types and conservation of lincRNAs. The gray lines in the outermost layer of the map represent lincRNA genomic clusters on each chromosome. The outer layer corresponds to the chromosome map of the human genome; black and white bars represent chromosomal cytobands and red bars represent centromeres. The dots in the map represent co-expression of lincRNA genomic cluster and positions of dots signify in 20 cancer types (BLCA, BRCA, CESC, THCA, GBM, SKCM, KICH, KIRC, KIRP, LGG, LIHC, LUAD, LUSC, OV, STAD, PRAD, HNSC, COAD, UCEC, READ). Line chart in the inner circles of the map represents the conservation of all lincRNA. The heatmap in the inner circles represents average expression levels of tumor samples of each expressed lincRNA in 20 cancer types (BLCA, BRCA, CESC, THCA, GBM, SKCM, KICH, KIRC, KIRP, LGG, LIHC, LUAD, LUSC, OV, STAD, PRAD, HNSC, COAD, UCEC, READ), only expressed lincRNAs (lincRNA expression is greater than 0.1 in more than two samples) were kept. (**B**) Ideogram showing the cluster size (the number of lincRNAs contained in the clusters) distribution on chromosomes. (**C**) Six examples of clusters containing cancer-related lincRNA genes are shown

We made a compromise choice to retain more lincRNAs and to improve the accuracy of lincRNAs clustering. Compared to 80 kb, 75% of lincRNA genomic clusters had been identified under 60 kb. In addition, clustered lincRNAs had consistent expression patterns at 60 kb. We defined 60 kb as the maximal distance for two lincRNA genes to be considered as clustered (Additional file 2: Table S2). By this definition, we constructed the circular map to obtain a global overview of the basic information, cluster locations, conservation scores, co-expression scores, and expression profiles in 20 cancer types for lincRNAs (Fig. 1A). We next wished to examine the number of lincRNA per cluster. The lincRNA genomic clusters in intergenic regions were found to be organized in 202 clusters including 126 quadruplets, 44 groups of five, 17 groups of six, 8 groups of seven, 4 groups of eight, one group of nine, two groups of ten. Figure 1B illustrated the chromosomal distribution of cluster size (the number of lincRNAs contained in the cluster).

In addition, some well-documented cancer-related lincRNAs have also been found clustered in the same clusters (Fig. 1C). For example, Cluster77 contains four lincRNAs, PCAT1 involved in prostate cancer development [40], PCAT2 associated with prostate cancer, PRNCR1 associated with include prostate disease and prostate cancer [41]. NEAT1 plays an oncogenic role in most types of solid tumors [42], and MALAT1 which is related to cancer pathophysiology [43] are both in Cluster93. Cluster116 is located on chromosome 13 and contains GAS6-AS2, LINC00454, LINC00452, and LINC00565. The long non-coding RNA GAS6-AS2 promotes bladder cancer proliferation and metastasis through the GAS6-AS2/miR-298/CDK9 axis. Aberrant expression of LINC00452 negatively correlates with recurrence-free survival time in ovarian cancer patients. The oncogenicity of LINC00452 is due to the formation of miR-501-3p sponges or physical effects that inhibit the Rho signaling pathway of key effector ROCK1 [44]. LINC00565 promotes ovarian cancer development by interacting with GAS6 as an oncogene [45].

### LincRNA genomic clusters shared common characteristics and potential biomedical significance

**Fig. 2:**
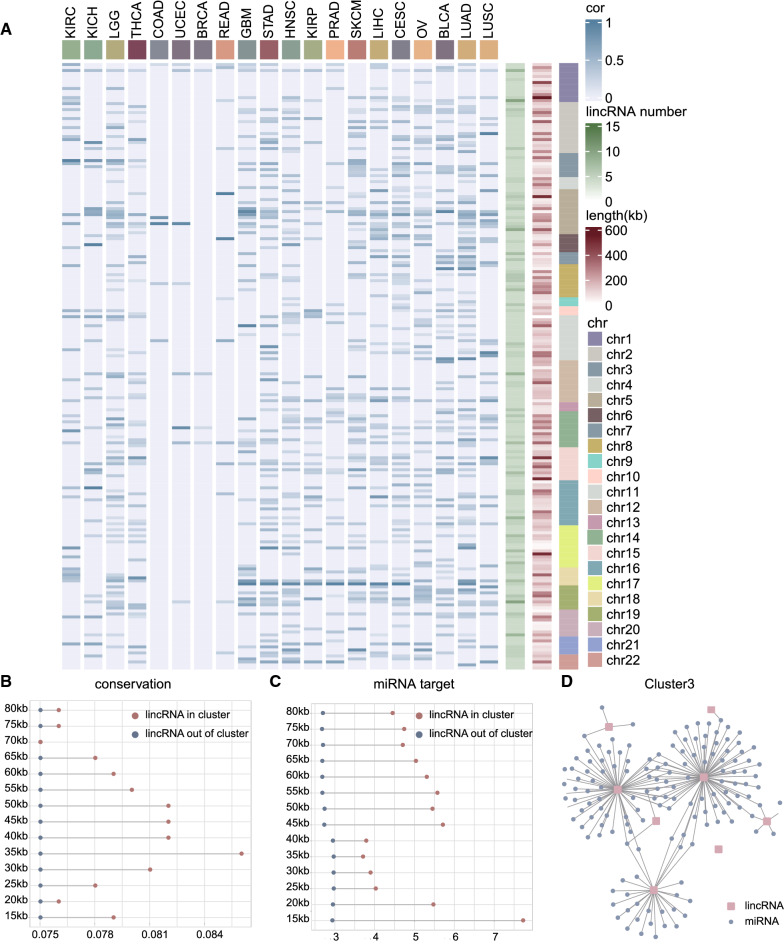
Common characteristics of lincRNA genomic clusters across cancer types. (**A**) Heatmap showing mean Pearson’s correlation coefficient of lincRNA genomic cluster in pan-cancer, cluster size, cluster length (distance between the start sites of the first lincRNA and the stop site of the last lincRNA in the cluster) and chromosome distribution. (**B**) LincRNAs_in_ conservation scores were higher than in lincRNAs_out_. (**C**) In lincRNAs_in_, numbers of miRNA target site were higher than in lincRNAs_out_. (**D**) MiRNA-lincRNA network of Cluster3 was constructed

We characterized the features of lincRNA genomic clusters from multiple perspectives across cancer types. First, we developed a new co-expression analysis framework to detect co-expression relationships between lincRNAs of each cluster in tumor expression profiles of 20 cancer types by integrating lincRNA annotation data and TANRIC lncRNA expression data (see “Materials and methods”). Co-expression modules were built by weighted gene co-expression network analysis (WGCNA) (Additional file 1: Fig. S3) and then we analyzed the mean Pearson's correlation coefficient of clusters in each cancer type (Fig. 2A). We found that out of 202 clusters, 168 clusters were strongly co-expressed among each other. Among all the clusters co-expressed, 7.73% of clusters co-expressed only in one cancer, and 10.1% of clusters were co-expressed in more than ten cancers (Additional file 1: Fig. S4). Interestingly, 63% lincRNAs of clusters co-expressed only in one cancer were distributed along chromosome 17 (Additional file 2: Table S3). Previous studies have reported a correlation between chromosome 17 and cancer [46]. This provided evidence that lincRNA genomic cluster may play a role in cancer.

Next, to define common characteristics of lincRNAs involved in the clusters (lincRNAs_in_), their related characteristics, including evolutionary conservation and miRNA targets were compared to those of lincRNAs not involved in the clusters (lincRNAs_out_). Previous reports have indicated that lincRNAs are generally less conserved than coding genes [47]. LincRNAs exhibit a range of conservation patterns, most of lincRNA sequences evolve very rapidly [1] while a minority of lincRNAs are highly conserved at both the sequence and the RNA secondary structure level [47]. The UCSC phastCons score provides a measure of evolutionary conservation based on multiple alignments of 99 vertebrate species. We used the UCSC phastCons score to assess the conservation of all lincRNAs. Next, we compared the conservation of lincRNAs_in_ and lincRNAs_out_ using Wilcoxon test. LincRNAs_in_ were more highly conserved than lincRNAs_out_ at different distance limit (P-value = 1.773E−04, Fig. 2B), indicating a lower selective pressure on these lincRNAs and highlighting the importance of their regulatory function. Then we used the UCSC 100-way phyloP score to compare the lincRNAs_in_ and lincRNAs_out_ under 60 kb. LincRNAs_in_ were more highly conserved than lincRNAs_out_ at 60 kb (Wilcoxon test, P-value = 0.034, phyloP (lincRNAs_in_) = − 0.154, phyloP (lincRNAs_out_) = − 0.160).

Recent studies have provided insights into the important influence of miRNA on lncRNA function [48]. We integrated diverse miRNA-lincRNA information from the four databases to find the relationship between miRNAs and lincRNAs. Last, lincRNAs_in_ had more miRNA binding sites (P-value = 3.321E−06, Fig. 2C), indicating complex regulatory functions among these lincRNAs. Most lincRNA genomic clusters shared the same miRNA binding sites, we next constructed a network of lincRNA-miRNA for each cluster. Cluster3, for example, 7 lincRNAs were targeted by 147 miRNAs (Fig. 2D), some cancer-related miRNAs such as hsa-miR-301a-3p [49], hsa-miR-34c-5p [50], hsa-miR-425-5p [51], and hsa-miR-4286 [52] interacting with two or more lincRNAs of Cluster3. Hsa-miR-301a-3p is involved in the epithelial–mesenchymal transition (EMT) process, which is thought to be involved in the development of laryngeal squamous cell carcinoma (LSCC). And hsa-miR-301a-3p acts as an oncogene by directly regulating the anti-oncogene Smad4, thereby playing a role in the occurrence and development of LSCC [49]. This result may suggest that the clustering lincRNAs interacting with several miRNAs could jointly involve in common biological processes, therefore shared more functional similarity. Thus, lincRNAs_in_ were greater than lincRNAs_out_ in terms of expression level, conversation scores, and density of miRNA binding sites, suggesting that they were more suited to play a role in biological processes.

We analyzed CNV data from TCGA across the 20 cancer types to assess CNV frequency of lincRNAs in each cluster. As shown in Additional file 1: Fig. S5, the loss frequency and the gain frequency were observed in lincRNA genomic clusters. The loss frequency ranged from 0 to 0.32, the gain frequency ranged from 0 to 0.38. LincRNAs in the same cluster tended to have similar loss and gain frequency.

For better detecting and characterizing the lincRNA genomic clusters shared among tumors, ssGSEA was performed based on lincRNA genomic clusters among 20 cancer types. Unsupervised hierarchical clustering of lincRNA genomic clusters ssGSEA scores was performed the modules of lincRNA genomic clusters (Additional file 1: Fig. S6). Four modules were identified, then we used lncSEA to perform enrichment analysis on the modules. We found that Module1 is related to three cancer hallmarks (prognosis, FDR = 4.00E−06; proliferation, FDR = 0.00332; migration, FDR = 0.0161).

### Conserved and cancer-specific lincRNA genomic clusters elucidated critical functions across cancers

**Fig. 3:**
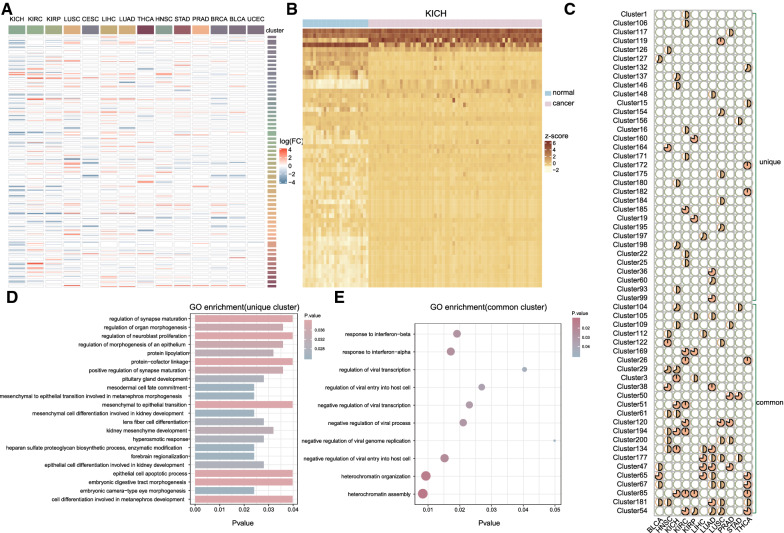
Co-differentially-expressed patterns of lincRNA genomic clusters in different cancer types. (**A**) Heatmap of differential expression among all clustered lincRNA in each cancer type. The color indicates different logFC (log2foldchange) values for lincRNAs from limma. Rows are arranged in the order of lincRNA genomic clusters. (**B**) Heatmap plot of significantly different expression of lincRNAs in co-differentially-expressed cluster in KICH. (**C**) The proportion of lincRNA in cluster which were differentially-expressed. (**D**) Annotation bars represent GO terms enriched for unique lincRNA genomic clusters ranked by P-value. The different colors showing the P-value of enrichment. (**E**) GO terms enriched for common lincRNA genomic clusters ranked by P-value are presented as bubble plots. The different colors showing the P-value of enrichment

Although lincRNA genomic clusters shared several common characteristics, analysis of the lincRNA genomic clusters across cancer types highlighted both common and specific features among cancers. For each cancer that had adjacent normal tissues, differential expression analysis was performed by comparing expression profiles of patient samples and normal samples to identify cancer-associated lincRNAs. Clusters containing more than half of the lincRNAs differentially expressed were considered to be co-differentially-expressed clusters. We performed pan-cancer analysis of differential expression based on 14 cancer types (Fig. 3A) and resulting in the identification of co-differentially-expressed clusters in 11 out of 14 cancer types. The expression heatmaps of all the lincRNAs in co-differentially-expressed clusters were shown in Fig. 3B and Additional file 1: Fig. S7. Among total of 202 clusters, 57 clusters co-differentially-expressed in one or more cancer types, including 33 clusters involved in a specific cancer type and 24 clusters involved in more than two cancer types. We referred to clusters involved in specific cancer types as "unique" and the group of clusters categorized as "common" (Fig. 3C), displayed differential dysregulation in multiple cancer types. Co-differentially-expressed frequencies of lincRNA genomic clusters were cancer type specific to a certain extent. For instance, 25% clusters were co-differentially-expressed in both kidney chromophobe (KICH) and kidney renal clear cell carcinoma (KIRC), but no co-differentially-expressed for breast invasive carcinoma (BRCA), cervical squamous cell carcinoma, and endocervical adenocarcinoma (CESC) and uterine corpus endometrial carcinoma (UCEC), indicating that many identified lincRNA genomic clusters had regulatory roles across multiple cancer types. In order to better find differential expression patterns of lincRNA genome clusters, the RNA-seq of tumor tissues and normal tissues of 20 cancer types were retrieved from TCGA and GTEx. We used limma to determine the differentially expressed clustered lincRNAs between normal and tumor tissues. As shown in Additional file 1: Fig. S8, the lincRNAs of the same lincRNA genomic cluster tend to be co-differentially expressed between TCGA and GTEx.

We further explored both common and unique features of lincRNA genomic clusters across the cancer types by performing functional enrichment analysis. LincRNAs in "unique" clusters were enriched in some GO terms related to epithelial-to-mesenchymal transition (EMT) in cancer such as "mesodermal cell fate commitment" and "mesenchymal to epithelial transition involved in metanephros" (Fig. 3D). Whereas functional enrichment analysis revealed that "common" clusters were mainly enriched in many defenses against virus processes and the regulation of transcription (Fig. 3E).

### Coordinate expressed lincRNA genomic clusters contributed to prognosis in human cancers

**Fig. 4:**
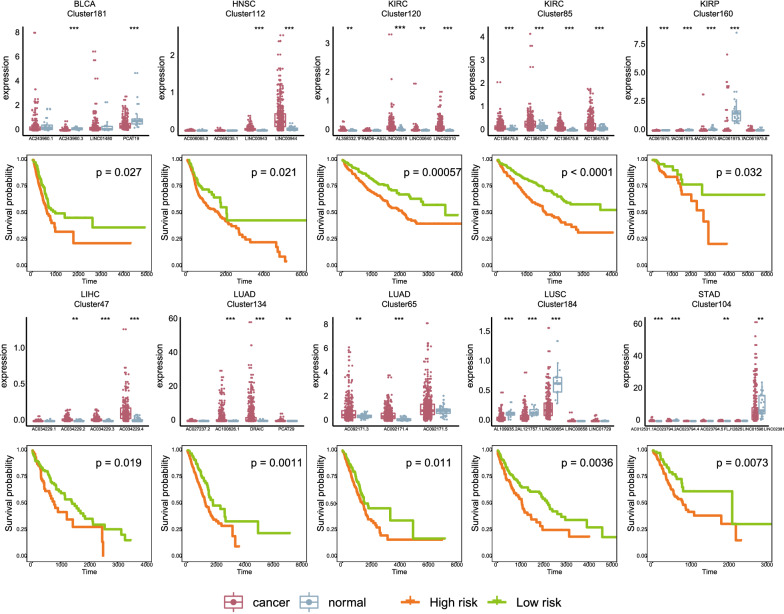
LincRNA genomic cluster as specific biomarkers of cancer. Survival analyses of 10 lincRNA genomic clusters in different cancer types were shown. The top panel showing the expression levels of lincRNAs in cancer and normal tissues. Patients are divided into low- and high-risk prediction groups based on the median risk score. The Kaplan–Meier curve showing the significant survival difference between the low- and high-risk groups in the prognostic model of each lincRNA genomic cluster. Survival days are shown along the x axis. Overall survival rates are shown along the y axis

Next, we examined whether the lincRNA genomic clusters were correlated with cancer survival and the expression of lincRNA genomic clusters could aid in distinguishing the two groups (see “Materials and methods”). It would be helpful to ascertain the potential of lincRNA genomic clusters as prognostic biomarkers with clinical implications. For each cancer type, we performed the Kaplan–Meier overall survival analysis for each clustered lincRNA. Next, all clusters were brought into further multivariate Cox proportional hazard regression analysis. As a result, in sixteen of the twenty cancers, lincRNA genomic clusters were significantly correlated with overall survival (P-value < 0.05, Fig. 4). Moreover, 85 clusters were correlated with survival, some clusters associated with poor prognosis of various cancers. For instance, Cluster54 contains LUCAT1 which is a biomarker for poor prognosis in NSCLC [53]. However, when expression profiles in the lincRNAs of Cluster54 were integrated and patients were reclassified into the high-risk group or low-risk group, survival analysis indicated that Cluster54 could distinguish the two groups of patients with different clinical outcomes. Moreover, Cluster54 was significantly related to survival in other six cancer types, lung adenocarcinoma (LUAD, P-value = 0.0066), brain lower grade glioma (LGG, P-value = 0.012), except kidney renal papillary cell carcinoma (KIRP, P-value = 0.015), KIRC (P-value < 0.0001), glioblastoma multiforme (GBM, P-value = 0.014), BLCA (P-value = 0.0028). Furthermore, Cox regression analysis indicated that not the individual lincRNA but the whole cluster was associated with patient survival in some clusters (Additional file 2: Table S4). These findings suggested the importance of coordinately expressed lincRNA genomic clusters in tumorigenesis and their prognostic value in clinical practice.

### LincRNA genomic clusters associated cancer biological functions

**Fig. 5:**
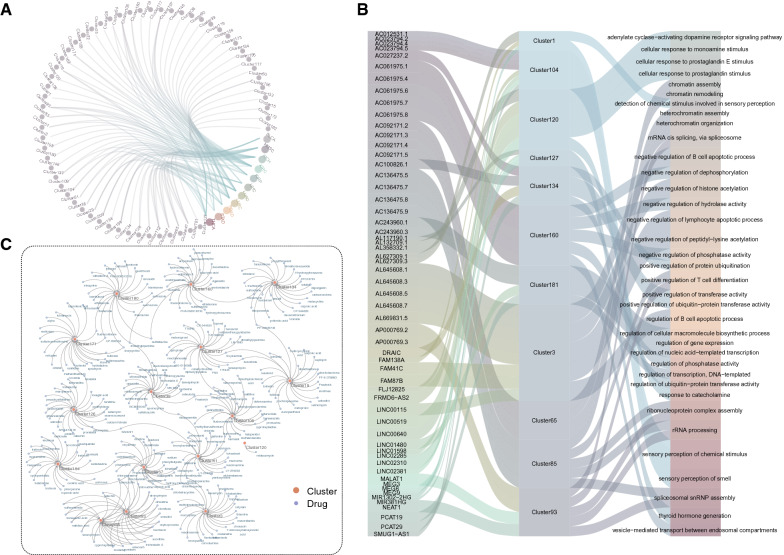
Functions of lincRNA genomic cluster in Pan-Cancer Analysis. (**A**) Cancer-cluster network was derived, which consisted of 57 highly synergistic expressed clusters and 11 cancer types. (**B**) The sankey diagram showing the correlation between lincRNAs contained in the clusters, lincRNA genomic clusters and GO terms. (**C**) The network was constructed by 16 clusters and CMAP drugs

Further, we analyzed the association of lincRNA genomic clusters with cancer. The co-expression and co-differentially-expression of each cluster in each cancer were shown in Additional file 1: Fig. S9. We considered clusters that were co-expressed and co-differentially-expressed in cancer as highly synergistic expressed clusters. 57 highly synergistic expressed clusters in 11 cancer types were identified, suggesting they may be co-regulated (Fig. 5A). To further explore each functional lincRNA genomic cluster, online Enrichr tool was employed for enrichment analyses of lincRNAs from each cluster based on Gene Ontology (GO) terms, KEGG pathways, and Connectivity Map (CMAP) drugs. Functional analyses revealed that some of them were enriched in various cancer-related biological processes (Fig. 5B). For instance, Cluster109 comprising four lincRNAs significantly enriched in several cancer-related biological processes such as regulation of MAPK cascade (GO:0043408) and Ras protein signal transduction (GO:0007265). Aberrant activation of RAS-MAPK pathway is a major and highly prevalent oncogenic event in many human cancers [54]. Survival analysis suggested that Cluster109 can be used as a potential prognostic biomarker for BLCA, KIRC, KIRP, LGG, LUAD, lung squamous cell carcinoma (LUSC), and Prostate adenocarcinoma (PRAD). In another example, Cluster164 consisting of four lincRNAs significantly enriched in several immune-related biological processes such as regulation of NK T cell activation (GO:0051133) and negative regulation of antigen receptor-mediated signaling pathway (GO:0050858). The immune system has many important regulatory roles in cancer development and progression [55]. Cancer prognosis may be related to immune system functional status [56]. Further, Cluster164 was correlated with survival in seven types of cancer including BLCA, BRCA, KIRP, LGG, LUSC, OV and SKCM. Some clusters were enriched in a number of cancer-related signaling pathways (Additional file 2: Table S5). Next, we built a network of drug–cluster interactions from CMAP drugs predicted by Enrichr (Fig. 5C, Additional file 2: Table S6). The network involved the construction of 16 lincRNA genome clusters and 285 small molecule drugs. The network showed that the clusters were targeted by multiple drugs and shared some drugs, suggesting these clusters may associate with the mechanism involved in the drug response.

### A lincRNA genomic cluster in imprinted *Dlk1-Dio3* locus associated with several cancers

To exemplify how these lincRNA genomic clusters provided insight into the pathogenesis of cancer, we examined a lincRNA genomic cluster mapping to *Dlk1-Dio3* imprinted locus. The *Dlk1-Dio3* imprinted cluster was located on chromosome 14q32.2, and the *Dlk1-Dio3* cluster had been shown to be imprinted in humans [57]. Deregulations of *Dlk1-Dio3* imprinted domain were involved in various diseases especially cancers [58]. The lincRNA genomic cluster Cluster127 was composed of MEG3, MEG8, MEG9, MIR381HG, LINC02285, AL132709.5, and AL132709.1. Among the cluster, previous researches have shown MEG3 [59] and MEG8 [60] significantly contribute to epigenetic EMT in the malignant progression of cancer. MEG9 protects endothelial cells from cell death induced by DNA damage.

**Fig. 6:**
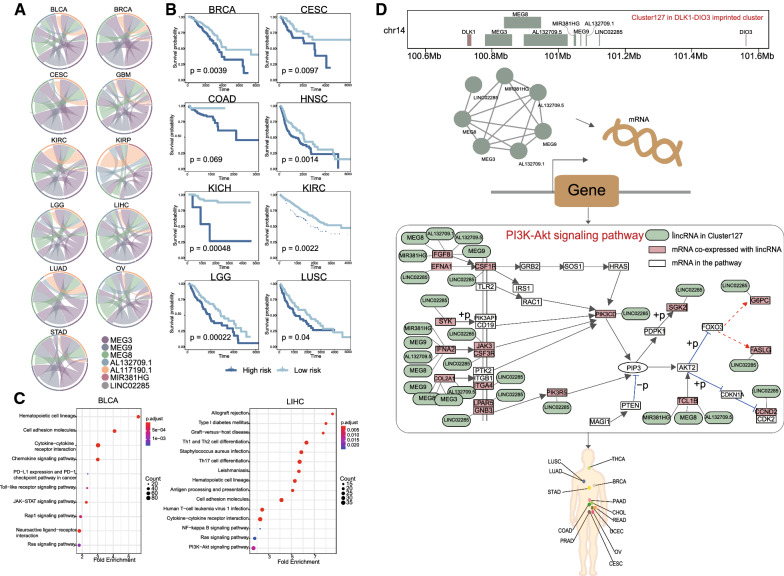
Cluster127 in imprinted *Dlk1-Dio3* locus is potential biomarker for cancers. (**A**) Expression relationships of the Cluster127 in 11 cancer types were displayed. (**B**) Kaplan–Meier plot for TCGA patients with different risk scores in cancers which Cluster127 is significantly associated with survival in BRCA, CESC, COAD, HNSC, KICH, KIRC, LGG, LUSC. (**C**) KEGG pathway analysis was applied for mRNAs co-expressed with Cluster127 in BLCA and LIHC separately. (**D**) A model illustrating the lincRNA of Cluster127 and mRNA co-expressed in LIHC cooperatively mediate the pathway dysregulation which may be implicated in cancer-related processes. LincRNAs in Cluster127 cooperatively mediated PI3K-AKT signaling pathway. The representative component of the pathway was shown. Protein coding genes co-expressed with lincRNA are indicated in red. The lines between them show the co-expression of lincRNA to genes. Cancers related to PI3K-AKT signaling pathway were linked with the literature reported

Next, we examined the relationship between the expressions of lincRNAs in the Cluster127 among cancers. This was important for evaluating the co-expression of qacluster. We tested whether gene pairs with high correlation (either positive or negative, cor = 0.35) showed an increased probability of coordinate function. The plot of the expression correlation revealed the expression of lincRNA in Cluster127 was highly correlated in tumor samples from different cancer types (Fig. 6A). We then performed multivariate Cox regression analysis to assess simultaneously the effect of lincRNAs in Cluster127 on survival time. The strong correlations between Cluster127 and survival supported their potential as specific cancer biomarkers in BRCA (P-value = 0.0039), CESC (P-value = 0.0097), HNSC (P-value = 0.0014), KICH (P-value = 0.00048), KIRC (P-value = 0.0022), LGG (P-value = 0.00022), and LUSC (P-value = 0.04) (Fig. 6B). Further Kaplan–Meier survival curve analysis indicated that all of those lincRNAs in Cluster127 did not affect survival rate, but the entire cluster correlated with overall survival in CESC and KICH (Additional file 1: Figs. S10 and S11), investigating their combined effects may regulate biological processes synergistically.

In addition, MEG3 can inhibit tumors by inducing the accumulation of p53 in cancer [61]. MEG8 inhibits the expression of genes that promote fibrosis and proliferation to inhibit epithelial–mesenchymal transition of hepatocytes. Taken together, these results suggested that these could regulate cancer development by targeting associated genes. Co-expression analyses of protein-coding RNAs and lncRNAs have been reported to study the potential function of lncRNAs in biological processes and cancers [62]. Furthermore, we further analyzed lincRNA-coexpressed mRNAs with more than six lincRNA co-expressed in 11 cancers. We finally identified that there are 1876 and 1461 co-expressed genes in BLCA and LICH respectively. Gene Ontology (GO) analyses of lincRNA-coexpressed mRNA genes in BLCA and LICH both revealed enriched categories of regulation of T cell activation, regulation of leukocyte cell–cell adhesion and T cell differentiation (Additional file 1: Fig. S12). KEGG pathway enrichment analyses were also performed on lincRNA-coexpressed mRNA genes in BLCA and LIHC (Fig. 6C). The lincRNA-coexpressed mRNAs genes in BLCA were most related to cancer-related pathways such as PD-L1 expression and PD-1 checkpoint pathway in cancer, Toll-like receptor signaling pathway, and JAK-STAT signaling pathway. The lincRNA-coexpressed mRNAs genes in LICH enriched some key pathways such as NF-kappa B signaling pathway and PI3K-AKT signaling pathway. PI3K-AKTpathway is an intracellular signaling pathway important in regulating the cell cycle. It has been shown that PI3K-AKT signaling pathway components are frequently altered in human cancers [63]. PI3K-AKT signaling pathway is involved in ovarian cancer (OV), uterine corpus endometrial carcinoma (UCEC), cervical cancer (CESC) [64], Renal Cell Carcinoma, Stomach adenocarcinoma (STAD) [65], colorectal cancer (COAD, READ) [66], breast cancer (BRCA) [67], lung cancer (LUSC, LUAD) [68], cholangiocarcinoma (CHOL) [69], prostate cancer (PRAD) [70], thyroid malignancies (THCA) [71], pancreatic cancer (READ) [72]. In the current study, 18 genes co-expressed with Cluster127 participated in this pathway (Fig. 6D). For example, COL2A1 was a key gene in PI3K-AKT signaling pathway and was also co-expressed with MEG9, MEG8, MEG3, and AL132709.5 in Cluster127. In summary, these results suggested that lincRNAs in Cluster127 could play their roles by participating in the regulation of PI3K-AKT signaling pathway.

## Discussion

Detecting deregulated lincRNAs is a major challenge of studying the mechanism and treatment about human cancer. It appears likely that lincRNA can coordinately exert their effects rather than individually. Moreover, several studies have suggested physically neighboring lincRNAs may work together. In the present work, a computational approach was developed to identify lincRNA genomic cluster based genomic locus.

In our analysis, lincRNA genomic clusters were found in the distance range of 15 to 80 kb. Here, we defined 60 kb as the maximal distance for two lincRNAs to be considered clustered. By this definition, the lincRNAs were found to be organized in 202 clusters. Next, we comprehensively evaluated the properties of lincRNA genomic clusters to obtain novel insights into synergistic effects at the lincRNA level in cancer. Permutation tests were additionally employed to ensure the reliability of properties.

We successfully determined differential expression based on lincRNA genomic clusters in a pan-cancer analysis. Co-differentially-expressed analysis showed that a proportion of lincRNA genomic clusters were conserved in cancers and varied greatly among diverse cancer types, other lincRNA genomic clusters were cancer-specific. Multidimensional genomics data provided more extensive insights into lincRNAs within the cluster functions and related pathways. Moreover, lincRNA genomic clusters might be effectively applied as new possible prognostic biomarkers for cancer. Especially, Cluster127 coordinated expressed in multiple cancers maybe contribute to the development of cancers. A key pathway named PI3K-AKT signaling pathway was found by functional analysis for genes co-expressed with Cluster127. Aberrant activation of the PI3K-AKT signaling pathway promoted the survival and proliferation of tumor cells in many human cancers.

In summary, a computational approach for the identification of lincRNA genomic clusters was constructed. We analyzed and characterized lincRNA genomic clusters of pan-cancer from many aspects. Our findings expanded the existing knowledge about the physical arrangement of lincRNA in relation to cancer. Integrating location and expression data of lincRNAs enhanced the interpretation capacity of lincRNA genomic clusters in cancers. These results could provide a new view of the characteristics and functions of lincRNA clusters based on genomic locus.

## Supplementary Information

**Additional file 1.** Additional figures.

**Additional file 2.** Additional tables.

## Data Availability

Not applicable.
