# Supplementary material for: Identifying and characterizing lincRNA genomic clusters reveals its cooperative functions in human cancer
Source: J Transl Med. 2021 Dec 14;19:509. doi: 10.1186/s12967-021-03179-5 (PMC8672572; doi:10.1186/s12967-021-03179-5)
Supplement: Supplementary file 1 — Additional file 1. Additional figures. [file 12967_2021_3179_MOESM1_ESM.docx]

**Identifying and characterizing lincRNA genomic clusters reveals its cooperative functions in human cancer**

*Zhou et al*

**Supplementary Figure**

**
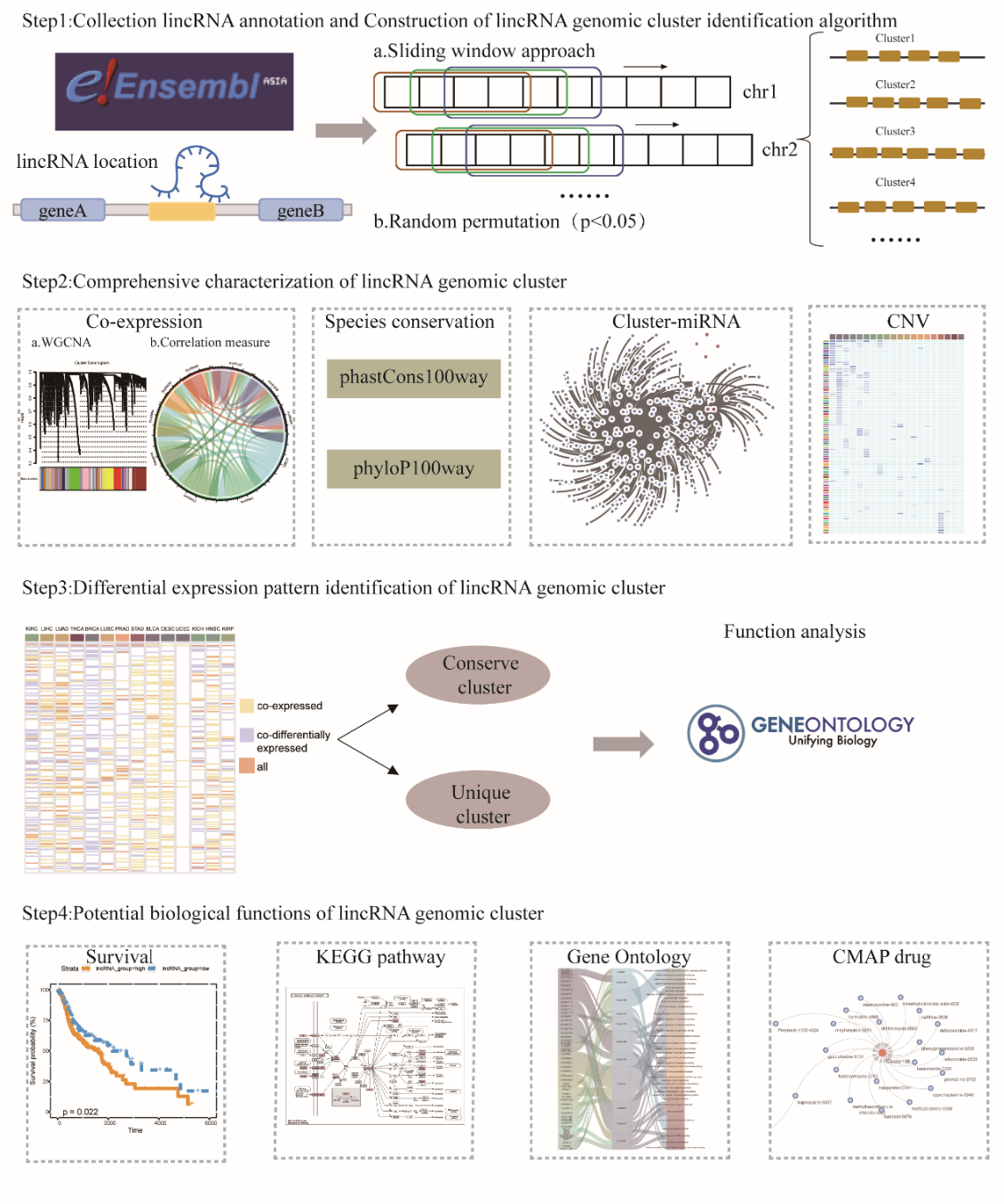
**

**Fig. S1**

**A comprehensive analysis workflow to characterize lincRNA genomic clusters.** Step 1: Collection lincRNA annotation and construction of lincRNA genomic cluster identification algorithm. We download the genomic location information of lincRNA from Ensembl. Next, we used the sliding window method to identify the lincRNA genome cluster from the beginning of each chromosome. We used random perturbation to calculate the significance of lincRNA genome cluster. Step 2: Comprehensive characterization of lincRNA genomic cluster. We performed comprehensive characterization of lincRNA genomic cluster from co-expression, species conservation, miRNA-lincRNA relationship and CNV pattern. Step 3: Differential expression pattern identification of lincRNA genomic cluster. Step 4: Potential biological functions of lincRNA genomic cluster. We characterize the function of lincRNA genome clusters using Survival analysis, KEGG pathway analysis, GO enrichment analysis and CMAP drug interactions.

**
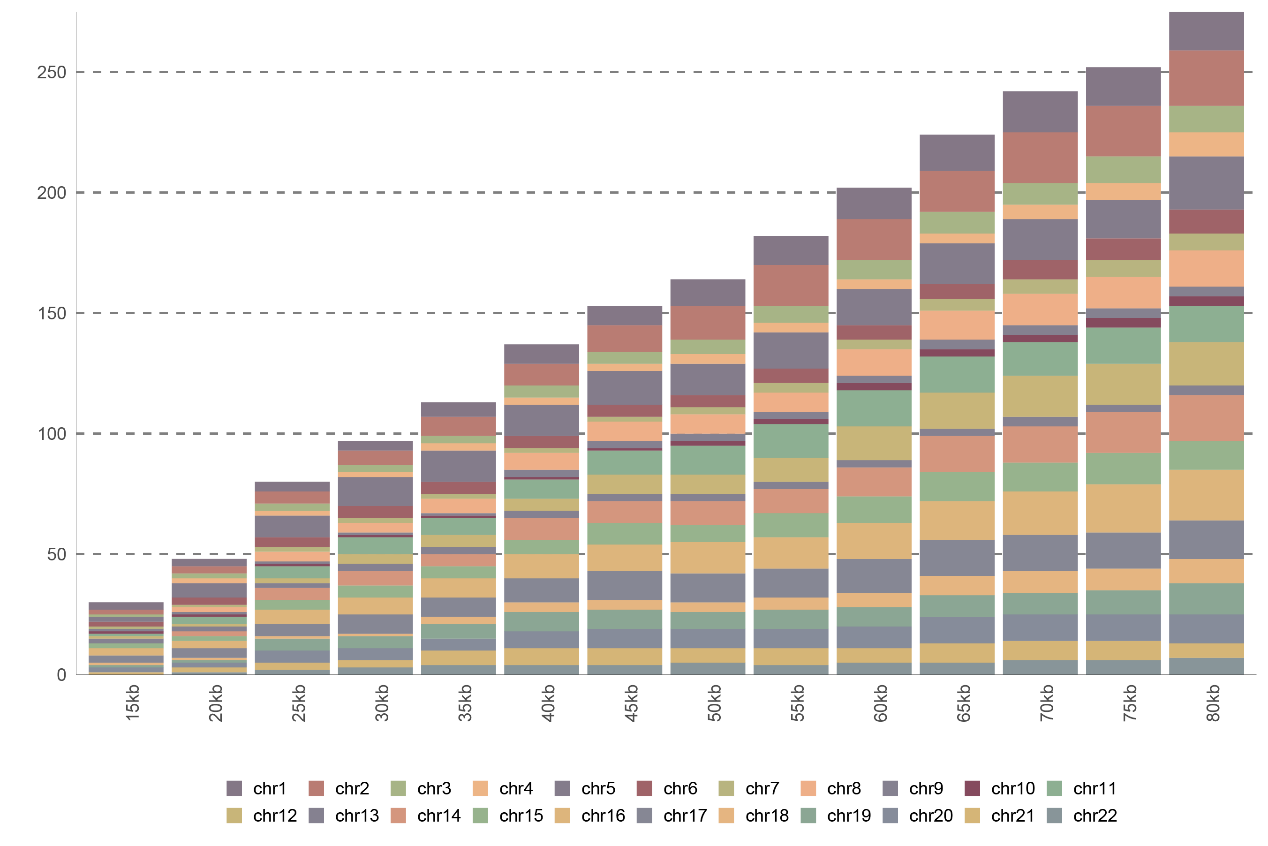
 Fig. S2**

**Distribution of lincRNA clusters on different chromosomes for different distances (15kb-80kb).**

**
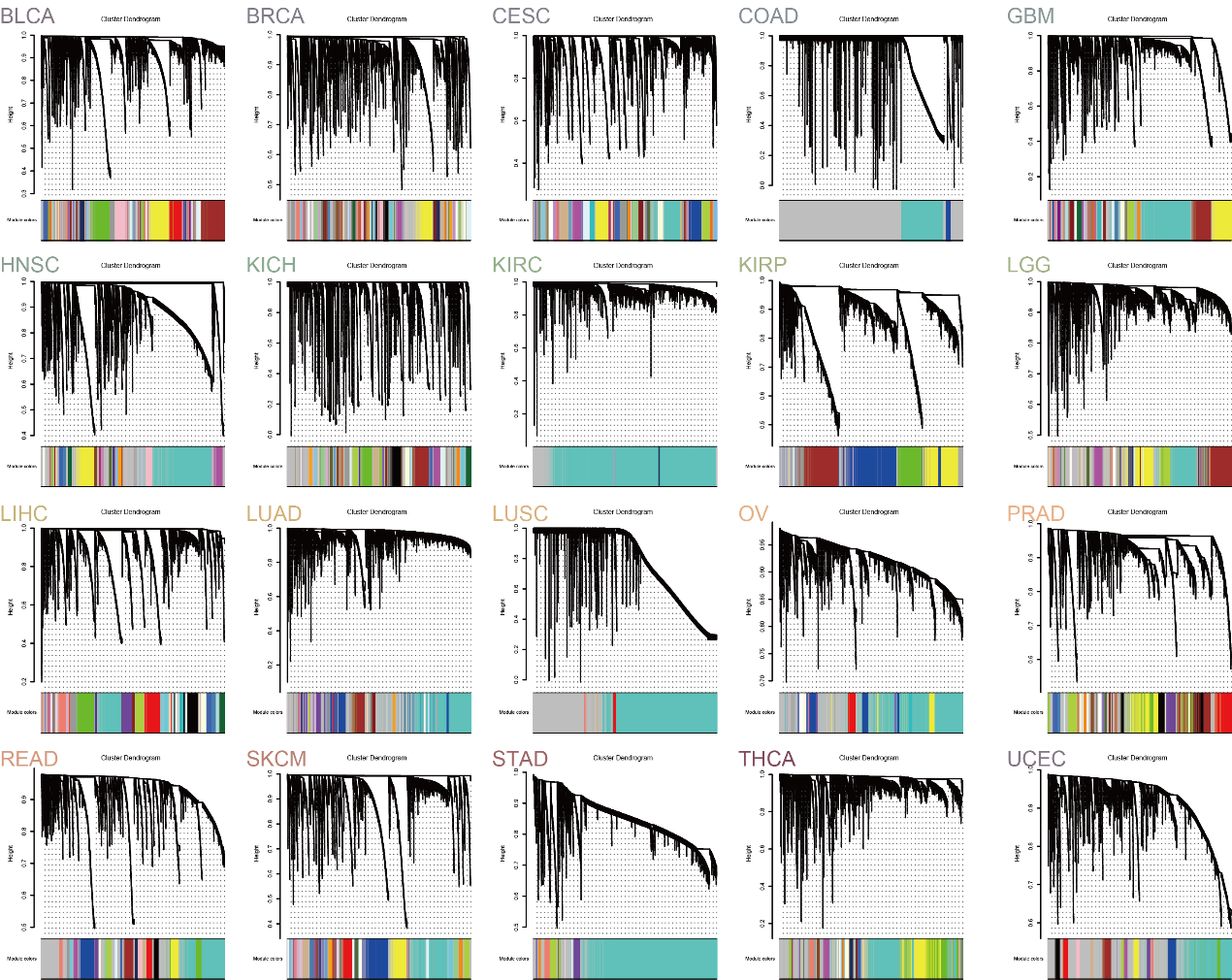
**

**Fig. S3**

**Cluster dendrogram of the coexpression network modules were produced based on topological overlap in the lincRNAs of different cancer types.**

**
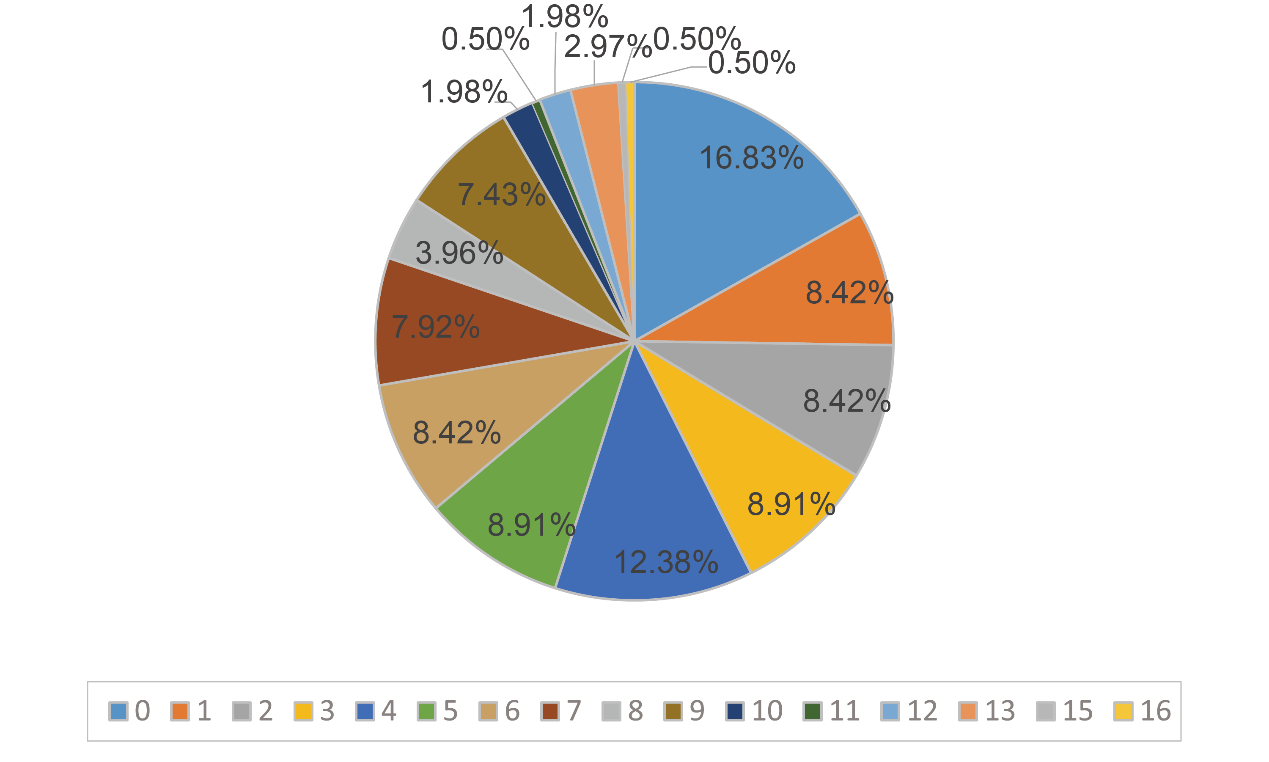
Fig. S4**

**The pie chart shows the proportion of co-expressed lincRNA clusters in different cancer types.**

**
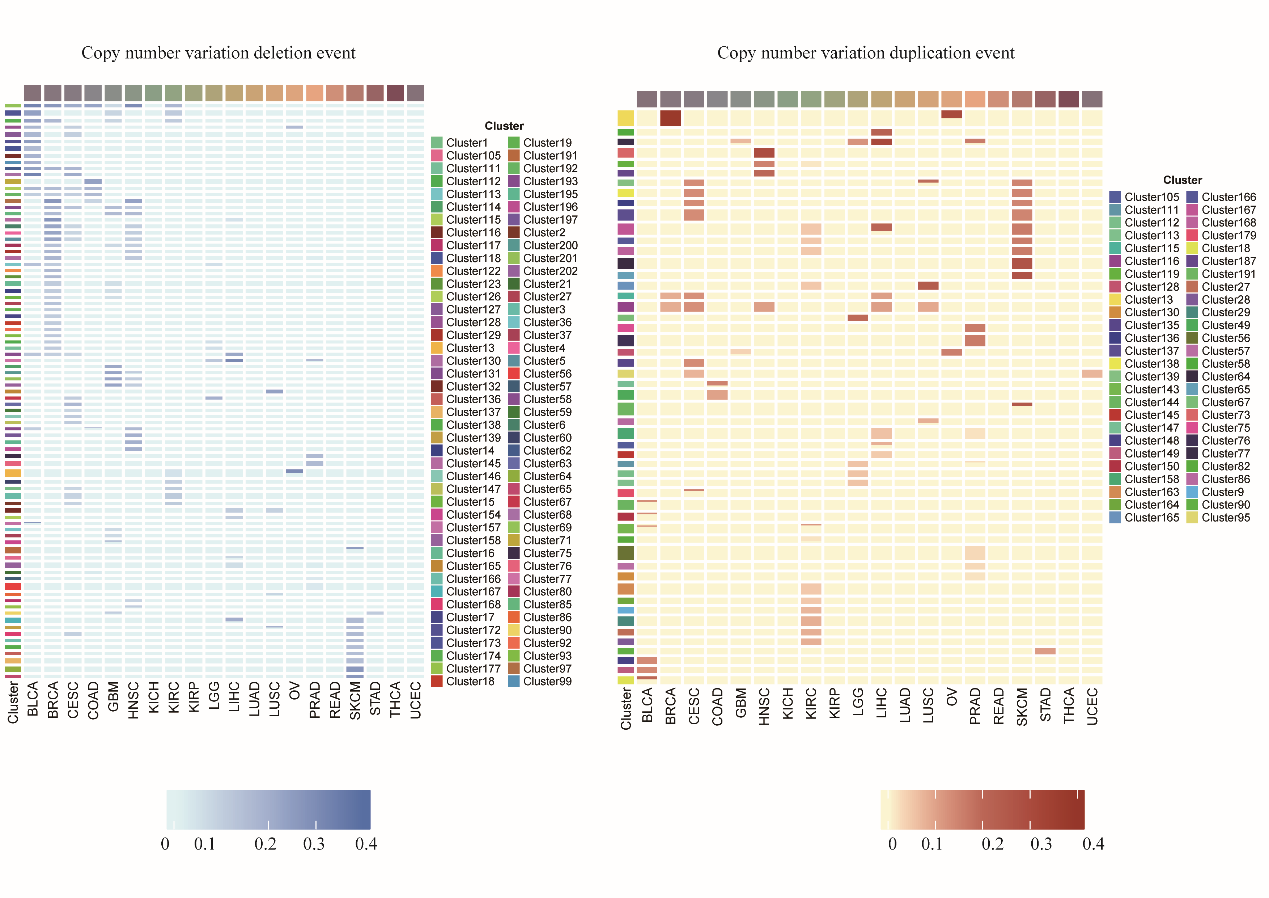
**

**Fig. S5**

**The CNV alteration frequency of lincRNA genomic clusters across cancer types.**


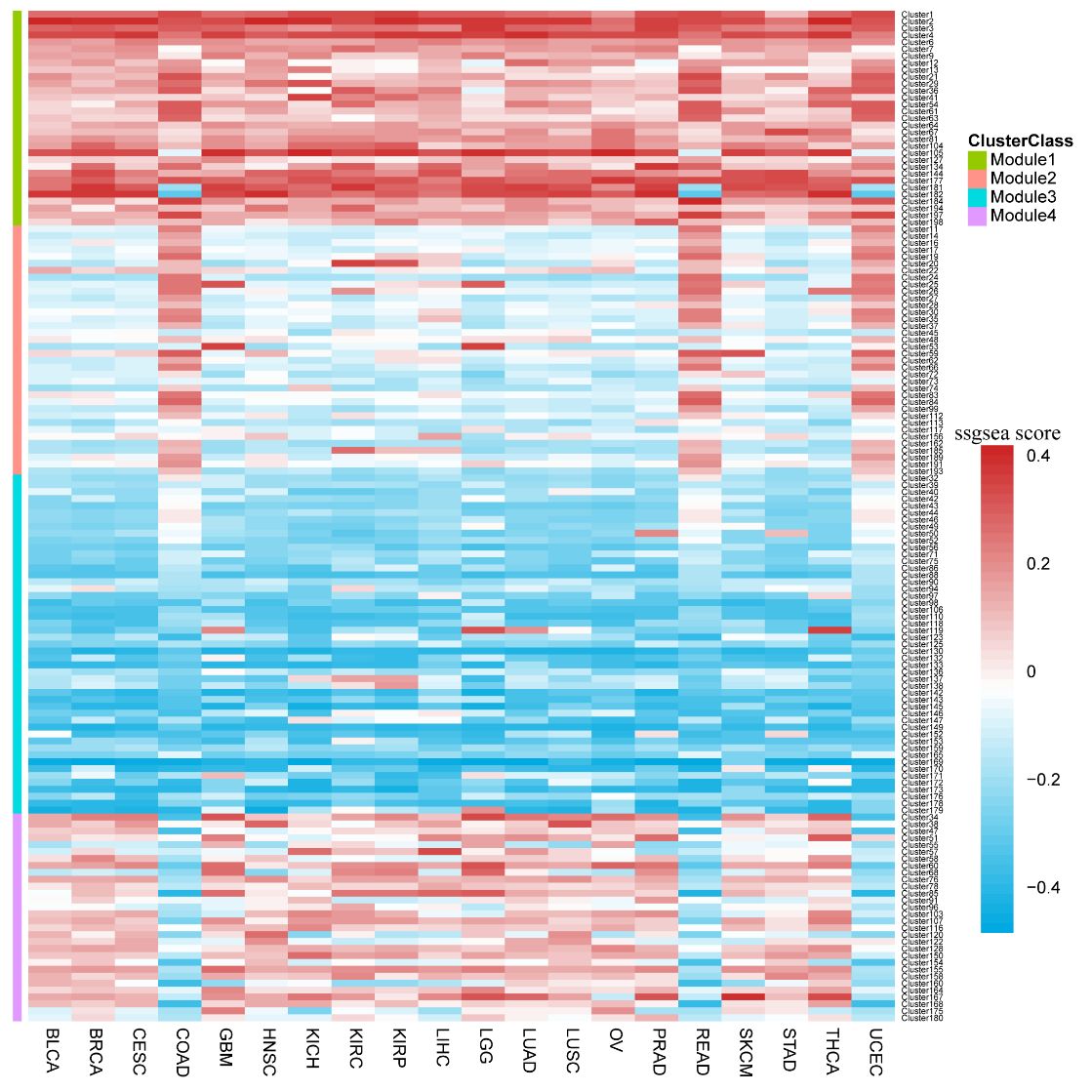


**Fig. S6**

**Heatmap of the four modules based on ssGSEA scores for lincRNA genomic clusters.**


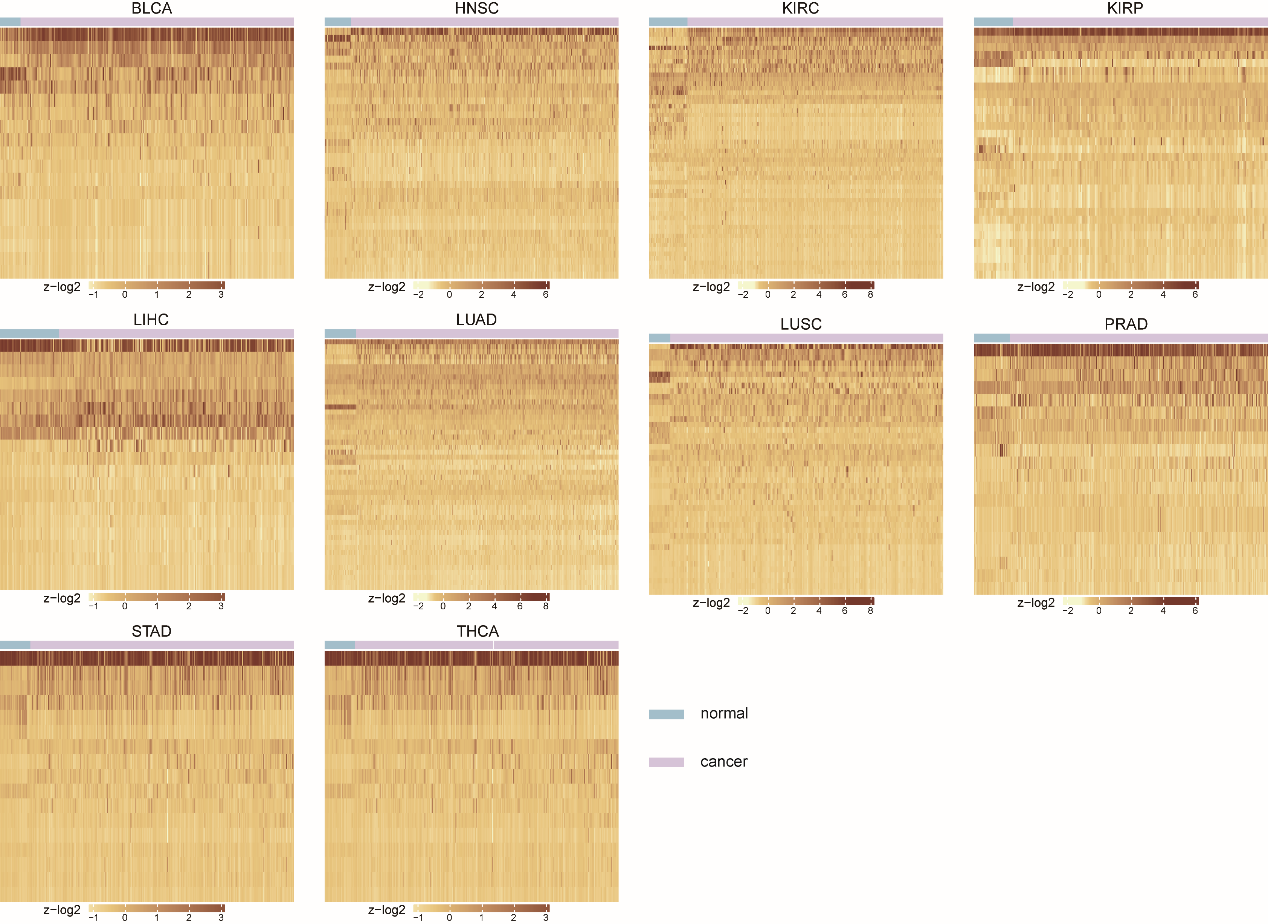


**Fig. S7**

**The expression profiles of lincRNAs in co-differentially-expressed clusters were displayed by heatmap among 10 cancer types.**

**
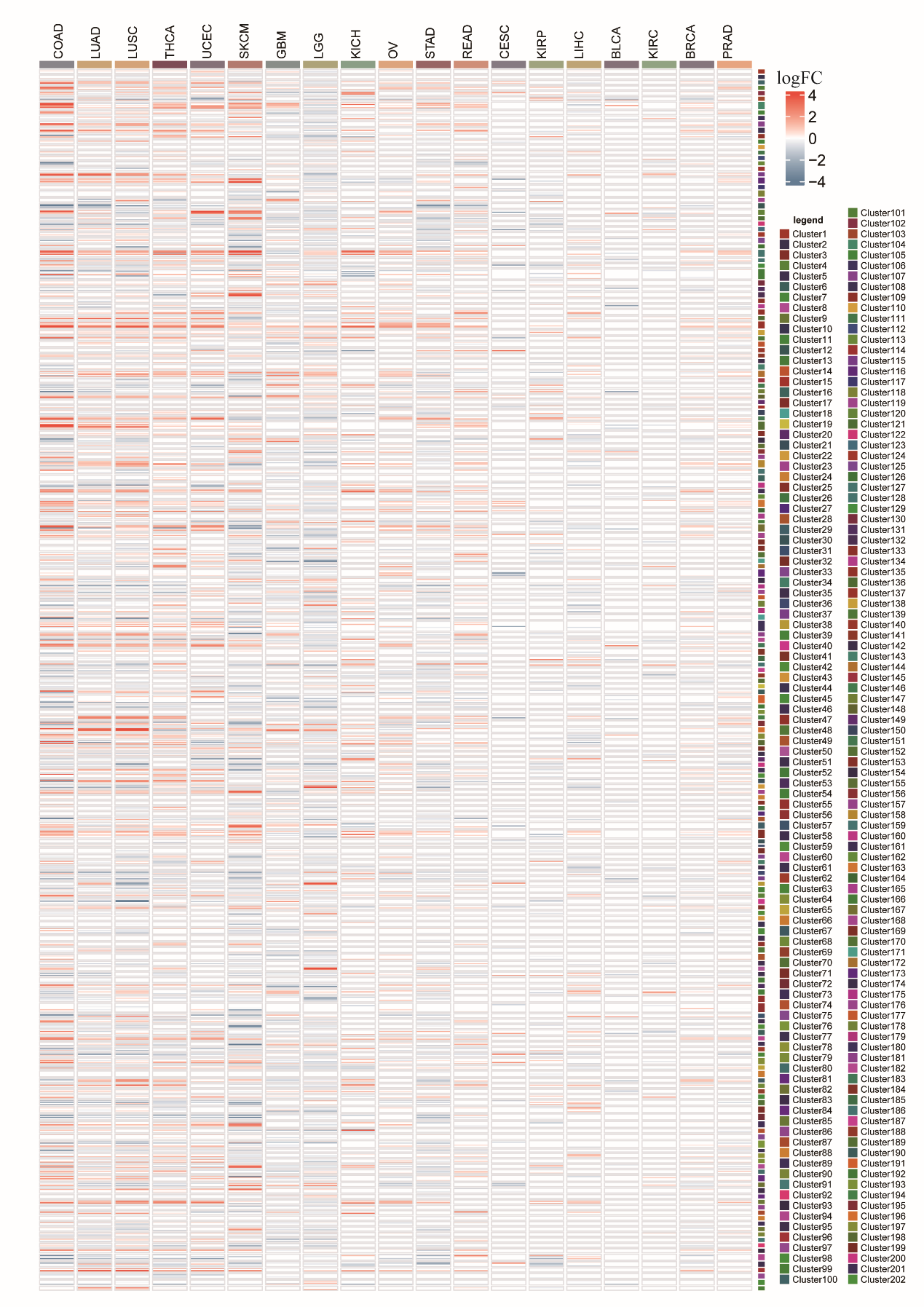
**

**Fig. S8**

**Differential expression heatmap of lincRNA genomic cluster between TCGA and GTEx.** The heat map showing the fold change of lincRNA differential expression. Annotation bars represent the lincRNA genomic clusters.

**
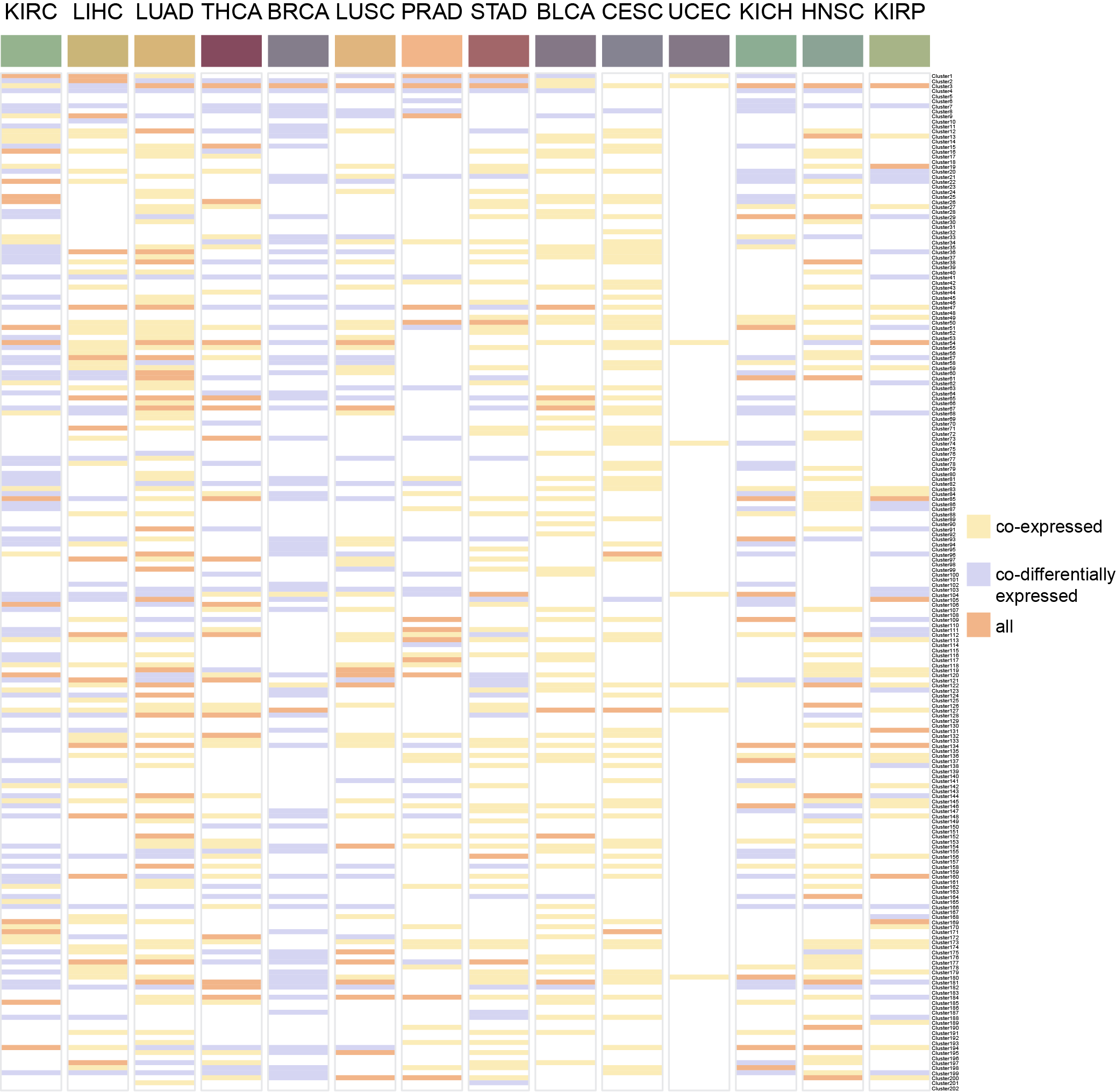
**

**Fig. S9**

**The co-expression and co-differentially-expression of each cluster in each cancer were shown by heatmap.**

**
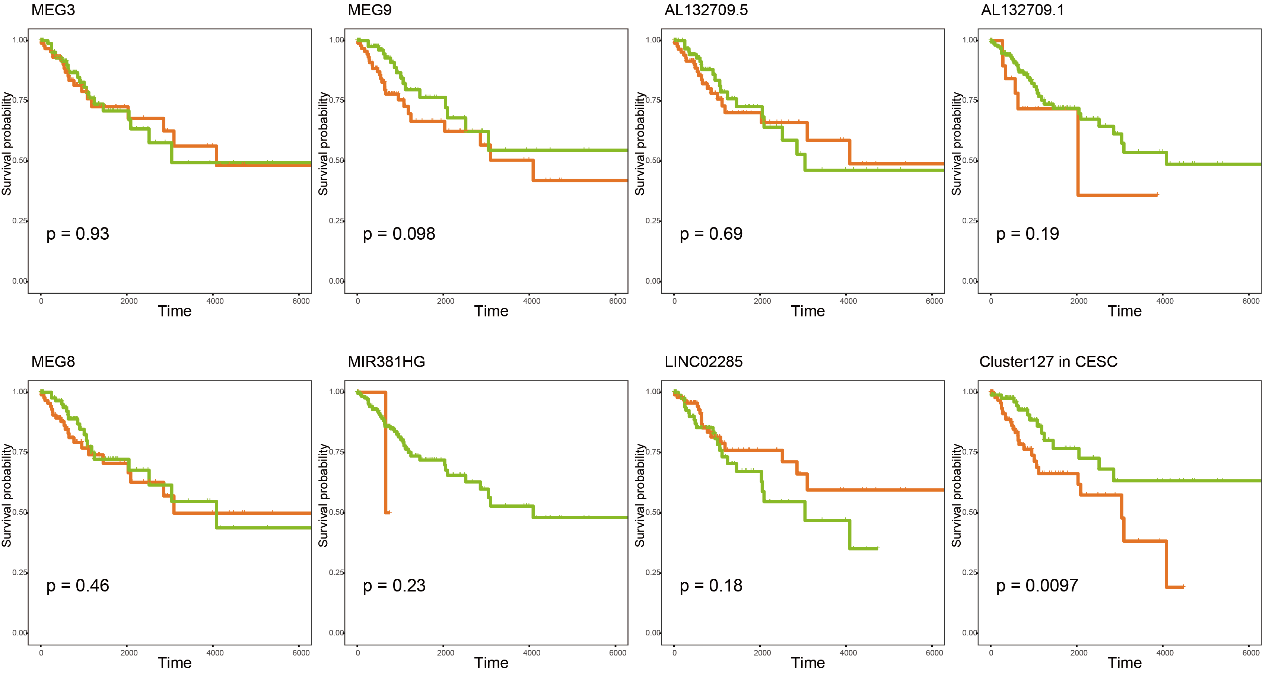
Fig. S10**

**Kaplan-Meier survival analysis of single lincRNA in the Cluster127**.

The single lincRNA could not distinguish CESC patients. Patients showing no progression or who were still alive at the time of the last follow-up were censored (+). P-values were calculated by the log-rank test. The multiple cox survival analysis revealed that Cluster127 could distinguish patients with different clinical outcomes in CESC.


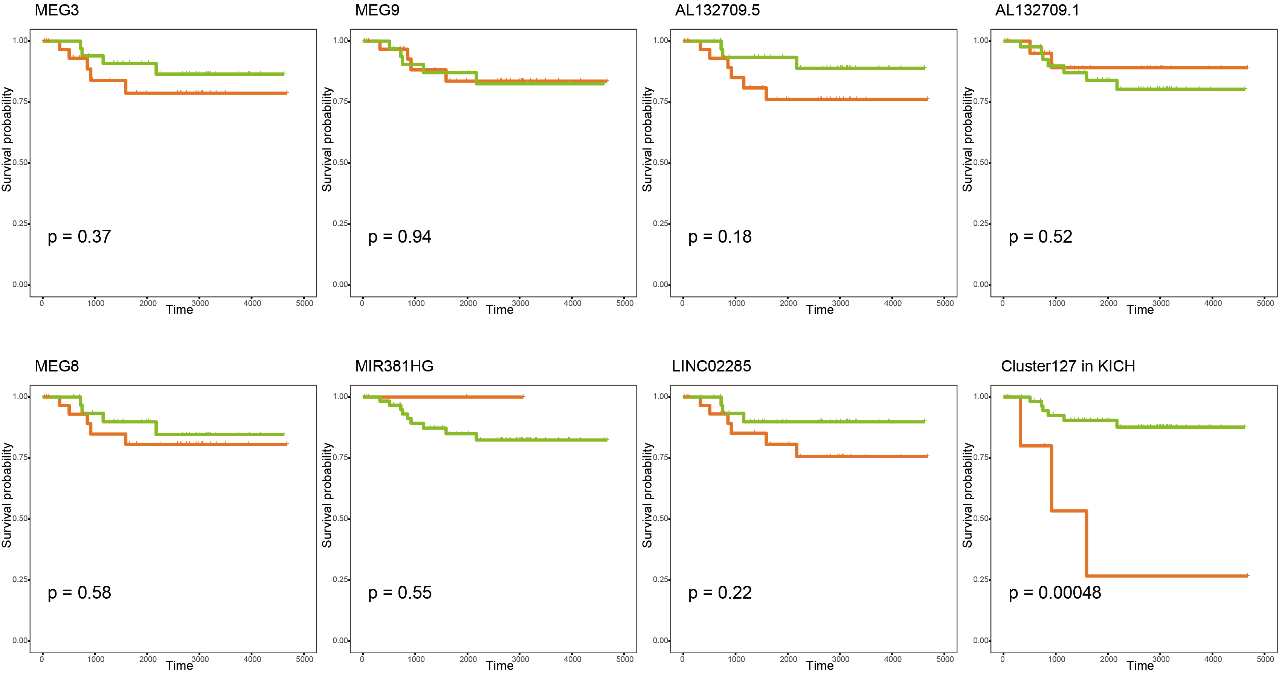


**Fig. S11**

**Kaplan-Meier survival analysis of single lincRNA in the Cluster127.** The single lincRNA could not distinguish KICH patients. Patients showing no progression or who were still alive at the time of the last follow-up were censored (+). P-values were calculated by the log-rank test. The multiple cox survival analysis revealed that Cluster127 could distinguish patients with different clinical outcomes in KICH.


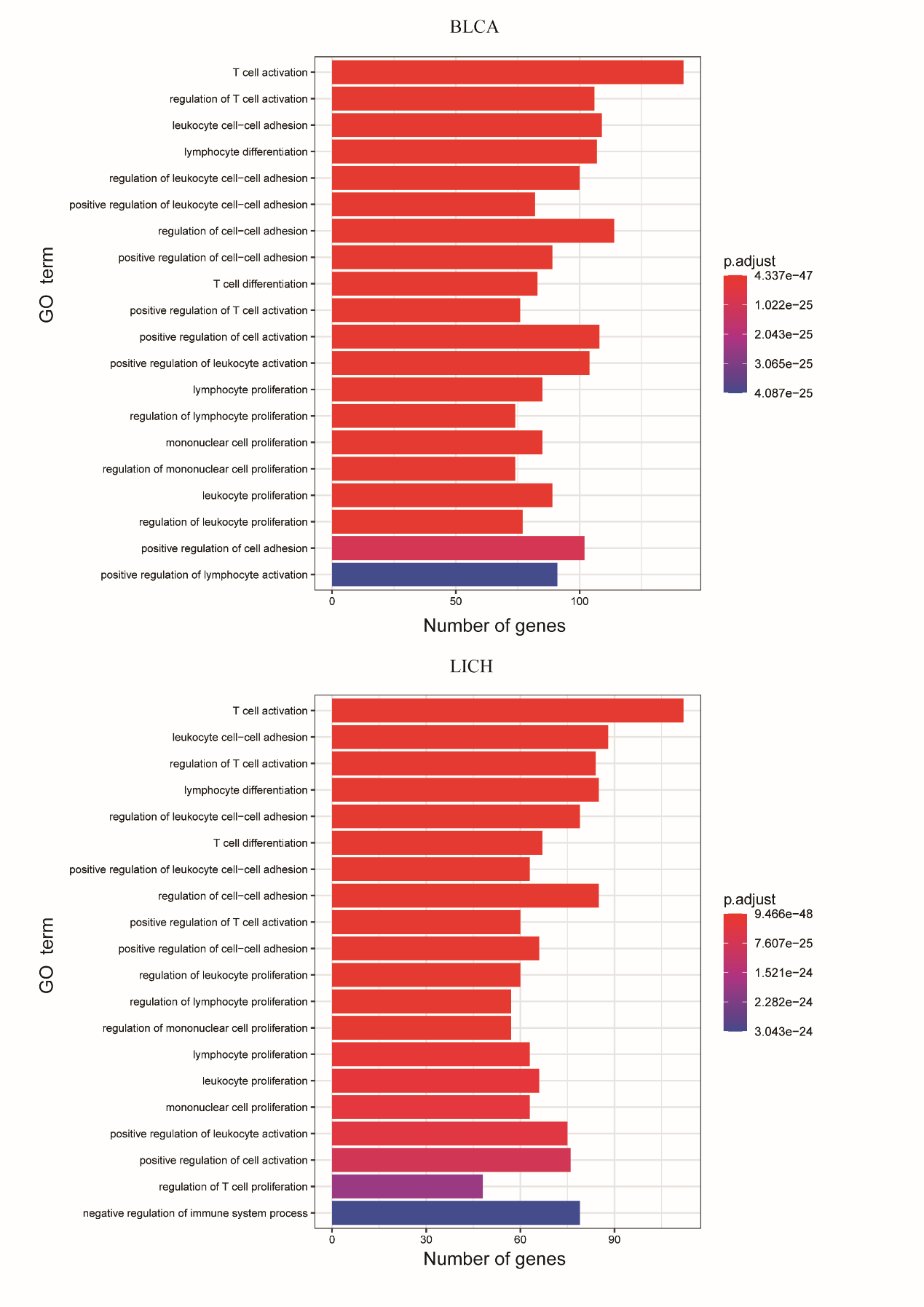


**Fig. S12**

**The GO enrichment analysis of mRNAs co-expressed with Cluster127 in BLCA and LIHC separately.** The different colors showing the adjust P-value of enrichment.
